# Differential expression and correlation analysis of whole transcriptome for type 2 diabetes mellitus

**DOI:** 10.3389/fendo.2025.1541261

**Published:** 2025-08-29

**Authors:** Fang Liu, Aihong Peng, Xiaoli Zhu, Guangming Wang

**Affiliations:** ^1^ School of Clinical Medicine, Dali University, Dali, Yunnan, China; ^2^ Hunan Clinical Laboratory Center, Changsha, Hunan, China; ^3^ Endocrinology Department, The First Affiliated Hospital of Dali University, Dali, Yunnan, China; ^4^ Center of Genetic Testing, The First Affiliated Hospital of Dali University, Dali, Yunnan, China

**Keywords:** type 2 diabetes mellitus, whole transcriptome sequencing, differential expression analysis, functional analysis, ceRNA network

## Abstract

**Background:**

Type 2 diabetes mellitus (T2DM) is a chronic metabolic disease that accounts for 90% or more of all diabetes cases and contributes to the global public health burden. The pathogenesis of T2DM is extremely complex, and increasing evidence suggests that non-coding RNA (ncRNA) is an important molecule involved in the regulation of T2DM. However, there are still many unknown lncRNAs and circRNAs that need further exploration. This study aims to explore new lncRNAs and circRNAs and their potential biological functions in T2DM.

**Methods:**

This study utilized high-throughput whole-transcriptome RNA sequencing technology to sequence and analyze five whole blood samples from each group, identifying differentially expressed mRNAs, lncRNAs, circRNAs, and miRNAs between the T2DM group and the control group. The biological functions of the differentially expressed RNAs were analyzed using Gene Ontology (GO) and Kyoto Encyclopedia of Genes and Genomes (KEGG) enrichment analysis. Subsequent association analysis was performed based on the screened differentially expressed mRNA, lncRNA, circRNA, and miRNA to construct a competitive endogenous RNA (ceRNA) network.

**Results:**

Differential expression results showed that 411 mRNAs were differentially expressed, 500 lncRNAs were differentially expressed, 356 circRNAs were differentially expressed, and 67 miRNAs were differentially expressed in patients with T2DM compared to controls. Functional analysis showed that cytokine−cytokine receptor interaction, graft−versus−host disease, inflammatory bowel disease, Lipid and atherosclerosis, sphingolipid signaling pathway, TNF signaling pathway, and FOXO signaling pathway, etc. play important roles in T2DM. The gene list was enriched with terms such as immune response, 1-phosphatidylinositol-3-kinase activity, oxidoreductase activity, action on the CH-NH2 donor group, interleukin-18 receptor activity, and antimicrobial peptide biosynthesis process, suggesting potential relevance to T2DM. In addition, six circRNAs and six lncRNAs were identified, which can compete with miRNA as ceRNA in the co-expression network.

**Conclusions:**

Differentially expressed circRNAs and lncRNAs may play a crucial role in T2DM. The ceRNA regulatory network provides new insights into T2DM.

## Introduction

Diabetes mellitus (DM) is a chronic metabolic disease that contributes to the global public health burden, with more than 536.6 million people aged 20–79 years living with diabetes globally in 2021 (with an estimated prevalence rate of 10.5%), and this number is projected to increase to 783.2 million (with an estimated prevalence rate of 12.2%) in 2045, according to IDF Diabetes (1). Of these, type 2 diabetes mellitus (T2DM), which accounts for 90% of all cases, is the most common type and is characterized by hyperglycemia and insulin resistance (2). The etiology and pathogenesis of T2DM are extremely complex and have not yet been fully elucidated. At present, it is believed that peripheral tissue insulin resistance and insulin secretion defects are mainly caused by genetic and environmental factors, leading to relatively insufficient insulin in the body. Epidemiological survey statistics show that obesity, overeating, and lack of exercise seriously affect its onset (3). An increasing body of evidence indicates that non-coding RNA (ncRNA) serves as a regulatory molecule involved in the pathophysiology associated with T2DM (4).

Traditionally, ncRNA was defined by its inability to encode proteins; however, recent studies have demonstrated that it is functional and regulates protein-coding gene expression via diverse mechanisms (5). Findings suggest that manipulation of ncRNA by functional non-coding RNA as regulators of specific gene expression may be a novel therapeutic approach to combat metabolic disorders such as diabetes (6). ncRNA mainly includes long non-coding RNA (lncRNA), circular RNA (circRNA), and microRNA (miRNA) (7). lncRNA is a non-coding RNA greater than 200 nucleotides in length, and it has been shown that lncRNA is involved in epigenetic regulation, transcription, translation, RNA metabolism, etc. (8). lncRNA can affect gene regulation in different ways: e.g., by binding to DNA-binding proteins (9); by recruiting epigenetic complexes, e.g., during DNA methylation (10); and by acting as precursors of small RNA, especially microRNA (11). circRNA is an endogenous biomolecule with a covalently closed transcription loop structure, expressed in various organisms (12). Reduced circHIPK3 and ciRS-7/CDR1as levels in pancreatic islets may disrupt β-cell function and affect insulin secretion and proliferation (13). In addition, circCAMSAP1 is abundant in human islets and expressed in peripheral blood (14), revealing the potential use of circRNA as biomarkers. Hsa_circ_0054633 was also found to be a diagnostic biomarker for prediabetes and T2DM (15). miRNA is a small and highly conserved class of non-coding RNA with regulatory functions found in eukaryotes. It controls post-transcriptional gene expression by degrading target mRNA or inhibiting protein translation (16). Many studies have shown that miRNA has important roles in the development of metabolic syndrome, e.g., in pancreatic β-cells, miRNA is important in maintaining the balance between differentiation and proliferation; miR-33a and miR-33b play key roles in cholesterol and lipid metabolism, and miR-103 and miR-107 regulate hepatic insulin sensitivity (17), and found that they also play important regulatory roles in immune disorders, cancer, and reproductive diseases (18).

Competitive endogenous RNA (ceRNA) is a non-coding RNA that competitively binds miRNA, which can effectively deregulate the inhibitory effect of miRNA on target genes, thereby increasing the expression of the corresponding mRNA to achieve the regulation of gene expression (19). ceRNA mainly include lncRNA, circRNA and pseudogenes (20). There is increasing evidence that many lncRNAs, circRNAs can act as ceRNA to influence the distribution of miRNAs in target genes.

Therefore, a comprehensive understanding of the molecular mechanisms of T2DM is important for accurate prediction and intervention. In this study, we used transcriptome sequencing technology to explore the differential expression of mRNA, lncRNA, circRNA, and miRNA between the T2DM group and the healthy control group. Through GO and KEGG enrichment analysis, the functional expression of abnormal target genes in type 2 diabetes was analyzed, and a ceRNA regulatory network was constructed to reveal the complex interactions among transcripts. It provides useful information for further understanding of its molecular mechanism, exploring potential therapeutic targets and prognosis.

## Materials and methods

### Patient and sample collection

In this study, we selected five patients newly diagnosed with type 2 diabetes mellitus (T2DM) who had not received treatment between March and September 2023 at the First Affiliated Hospital of Dali University as the experimental group and five healthy individuals who underwent physical examinations at the same hospital during the same period as the control group. The basic information of the study subjects is shown in **Tables 1–1**, **1–2**. All patients met the diagnostic criteria for T2DM (21). And patients with acute infectious diseases, coronary atherosclerotic heart disease, atrial fibrillation, myocardial infarction, immune diseases, and a history of hematologic diseases were excluded. The ethical implications of the study were approved by the Ethics Committee of the First Affiliated Hospital of Dali University (Ethics No. DFY20220415001), and informed consent was obtained from all participants.

**Table 1-1:** Descriptive characteristics of participants.

Characteristics	Case	Case	Case	Case	Case	Control	Control	Control	Control	Control
	1	2	3	4	5	1	2	3	4	5
Gender	Woman	Man	Man	Woman	Man	Man	Man	Man	Woman	Woman
Age	55	60	61	53	44	61	54	55	53	52
Fasting blood glucose	6.16	6.23	6.88	11.31	11.95	4.61	4.89	4.99	4.79	4.69
HbA1c	6.5	5.8	7	14.9	14.4	4.8	5.1	5.5	5.0	5.4
DM	Yes	Yes	Yes	Yes	Yes	No	No	No	No	No
Hypertension	No	No	Yes	No	No	No	No	No	No	No
CHD	No	No	No	No	No	No	No	No	No	No
Hyperlipidemia	Yes	No	No	Yes	Yes	No	Yes	No	No	Yes

**Table 1-2:** OGTT test for patients in the experimental group.

Glucose Measures	Case1	Case2	Case3	Case4	Case5
Fasting blood glucose	7.13	6.37	7.87	8.83	8.37
OGTT 1h blood glucose	14.43	10.77	10.33	17.7	10.86
OGTT 2h blood glucose	12.59	12.01	14.52	20.7	14.69

Immediately after the patients had fasted for 12 hours, 5 mL of peripheral blood was collected from the study subjects using a BD PAXgene Blood RNA Tube (PAXgene) blood collection tube, and after the collection was completed, the exterior of the blood collection tube was sterilized with 75% medical alcohol, and then the PAXgene tube was stored for spare use.

### RNA extraction

Total RNA was extracted from blood samples using the SteadyPure Blood RNA Extraction Kit in strict accordance with the requirements. The purity of RNA was detected using a Keio K5500 spectrophotometer (Kayo, Beijing, China), and the concentration and integrity of RNA were detected using an Agilent 2100 RNA Nano 6000 Assay Kit (Agilent Technologies, CA, USA). In this study, the quality standards for RNA library construction sequencing are as follows: when the concentration is ≥100 ng/μL, the total amount is ≥1 μg, the OD260/280 ratio is between 1.8 and 2.0, and the RIN value is ≥5.8, the sample meets the requirements for library construction sequencing.

### Library construction and transcriptome sequencing

We built two sequencing libraries. The NEBNext^®^ Ultra™ Directional RNA Library Prep Kit for Illumina^®^ (NEB, USA) was used for lncRNA, circRNA, and mRNA sequencing. Take 3μg of total RNA from each sample as the starting amount to construct an RNA library. The rRNA was first removed from the samples using Ribo-Zero™ Gold Kits, and Fragmentation Buffer was added to the reaction system to fragment the RNA. Then the fragmented RNA was used as a template to synthesize the first strand of cDNA with six-base Random Hexamers, followed by the addition of buffers, dNTPs, RNAse H and DNA Polymerase I to synthesize the second strand of cDNA, and then purified by the QiaQuick PCR kit, eluted with EB buffer, and amplified by end repair, addition of base A, addition of sequencing junction, fragment selection, digestion of cDNA second strand with UNG enzyme, and purification and enrichment of the product by polymerase chain reaction (PCR) to amplify the library DNA. Finally, the library was sequenced on the Illumina NovaSeq 6000 platform.

The NEBNext^®^ Multiplex Small RNA Library Prep Set for Illumina^®^ (NEB, USA) was used for miRNA sequencing. After the total RNA samples were tested and qualified, the total RNA was firstly subjected to fragment selection, and the RNA fragments of 18–30 nt or 15–35 nt were collected by gel separation technique; the 3’ and 5’ junction were connected at the ends of the isolated RNA fragments, and then reverse transcribed into cDNA, and then PCR amplification was performed to establish the sequencing libraries. The qualified sequencing library was sequenced by the Illumina HiSeq X platform.

Illumina high-throughput sequencing raw image data files were converted to raw sequencing sequences stored in FASTQ file format after base identification by bcl2fastq software. The raw downlink data sequences are filtered to obtain high-quality Clean Reads for subsequent analysis. Then, HiSAT2 was used to compare and analyze the filtered RNA-seq data of each sample with the genome. Rapid assembly of transcripts using StringTie software. lncRNA sequencing can simultaneously obtain mRNA and lncRNA information, followed by comparative analysis of lncRNA and mRNA based on the transcript results. lncRNA refers to long non-coding RNA with a length greater than 200 bp. Based on their positional relationship with coding sequences, they are classified into types such as lincRNA, intronic lncRNA, anti-sense lncRNA, sense lncRNA, and bidirectional lncRNA. Among these, lincRNA accounts for the highest proportion. When screening for novel lncRNA, the focus is primarily on lincRNA, intronic lncRNA, and anti-sense lncRNA. After setting the screening criteria, the lncRNA identified through screening is used as the final candidate novel lncRNA for subsequent analysis.

To obtain circRNA, raw sequencing data were analyzed using CASAVA software to obtain clean reads. The BWA-MEM algorithm in BWA was used to align the reads with the genome and the CIRI tool was used to identify circRNA. DEseq2 was used for circRNA differential expression analysis to compare the treatment group with the reference group, and |log2foldchange|≥1 and p-value<0.05 were selected as significantly differentially expressed circRNA to obtain the number of significantly up- and down-regulated circRNA.

To obtain miRNA, raw data in fastq format were first processed by bcl2fastq. Raw Reads were processed with cutadapt for splice removal, low quality removal, and fragment selection to obtain clean reads for subsequent analysis. For the accuracy of the subsequent analysis, the clean reads from sRNA sequencing were first localized to the reference genome by the comparison analysis software Bowtie, and the number of Total Clean Reads on the comparison and its comparison rate were counted. Based on the reads of the reference sequences on the comparison, the sequences of the specified species were compared with the sequences in the miRBase database to get the different regions of sequence matching in each sample.

### Identify differentially expressed mRNA, lncRNA, circRNA and miRNA

The DEseq R package was used to analyze the differential expression of mRNA, lncRNA, circRNA, and miRNA between the type 2 diabetes group and the control group. The screening threshold was set at p < 0.05 and |log2FC| ≥ 1 to obtain the most significantly differentially expressed mRNA, lncRNA, circRNA, and miRNA. The volcano plotting and heat mapping were performed using the R packages “ggplot2” and “pheatmap,” respectively.

### GO and KEGG enrichment analysis

To better understand the mechanism of action of T2DM, we annotated the functions of differentially expressed RNAs using GO and KEGG enrichment analysis based on transcriptome sequencing analysis of mRNA, lncRNA, circRNA, and miRNA. GO consists of three ontologies describing the molecular functions, cellular components, and biological processes of genes, providing comprehensive gene annotation information, while KEGG enrichment analysis helps us to understand the biological processes, signaling pathways, and other functions that these genes may activate. We selected p-value < 0.05 and FDR value < 0.05 as the criteria for significantly enriched GO terms. Enrichment analysis was performed by applying the hypergeometric test to each pathway in KEGG to identify the pathways that were significantly enriched for differentially expressed genes.

### Construction of relevant regulatory networks

Based on the ceRNA theory, we constructed a ceRNA regulatory network by integrating the expression profiles and regulatory relationships of lncRNA, circRNA, miRNA, and mRNA. The ceRNA network, composed of lncRNA-miRNA pairs, circRNA-miRNA pairs, and miRNA-mRNA pairs with the same miRNA node, was visualized using Cytoscape 3.9.1 software. Different shapes were used to distinguish between various types of RNAs, while colors were used to differentiate between up-regulated and down-regulated RNAs.

### Statistical analysis

The statistics were analyzed using R software. Differences in the expression of lncRNA, circRNA, and mRNA between the T2DM group and the control group were analyzed using the Student’s t-test; *p* < 0.05 was considered statistically different.

## Results

### Overview of the transcriptome profiling

Before further analysis, we need to ensure the accuracy of the results. In the circRNA, lncRNA, and miRNA libraries, 75818172, 75889210, 92141250, 82224200, and 93285246 clean reads were generated in five T2DM patients, respectively; then, in the 5 healthy control groups, 97370628, 95551370, 83143362, 95994314, and 94235596 clean reads were generated, respectively. Detailed quality control results are listed in **Supplementary Table 1**.

### Differential expression profiles of mRNA, lncRNA, circRNA, and miRNA between the T2DM group and healthy controls

**Figure 1:**
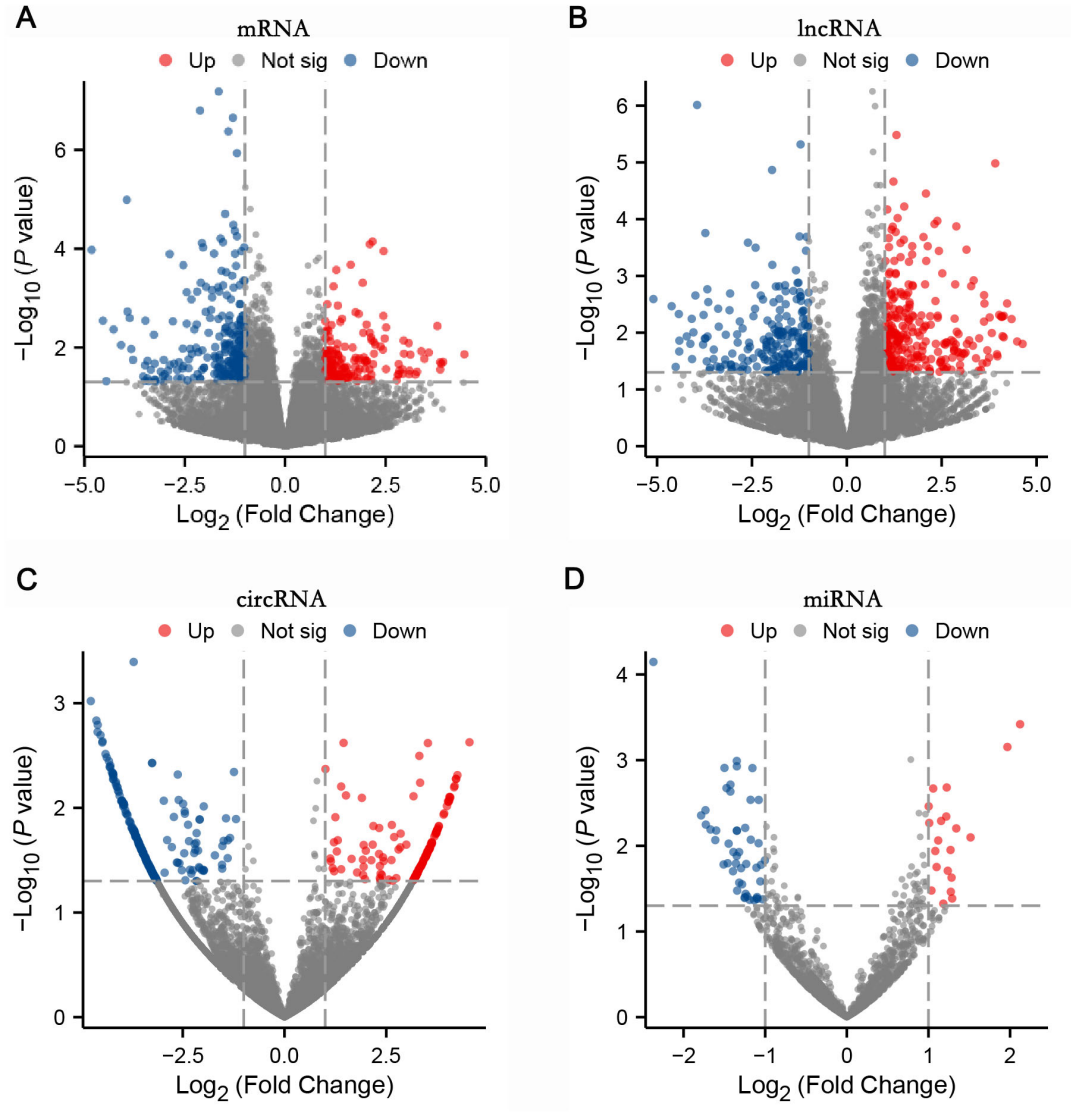
Four volcano plots: **(A)** mRNA, **(B)** lncRNA, **(C)** circRNA, and **(D)** miRNA showed differential expression analysis. The X-axis represents log2 fold change, and the Y-axis represents negative log10 p-value. Red dots indicate upregulated, blue indicate downregulated, and grey indicate not significant. Each plot displays data points distributed around a central region with varying significance levels.

**Figure 2:**
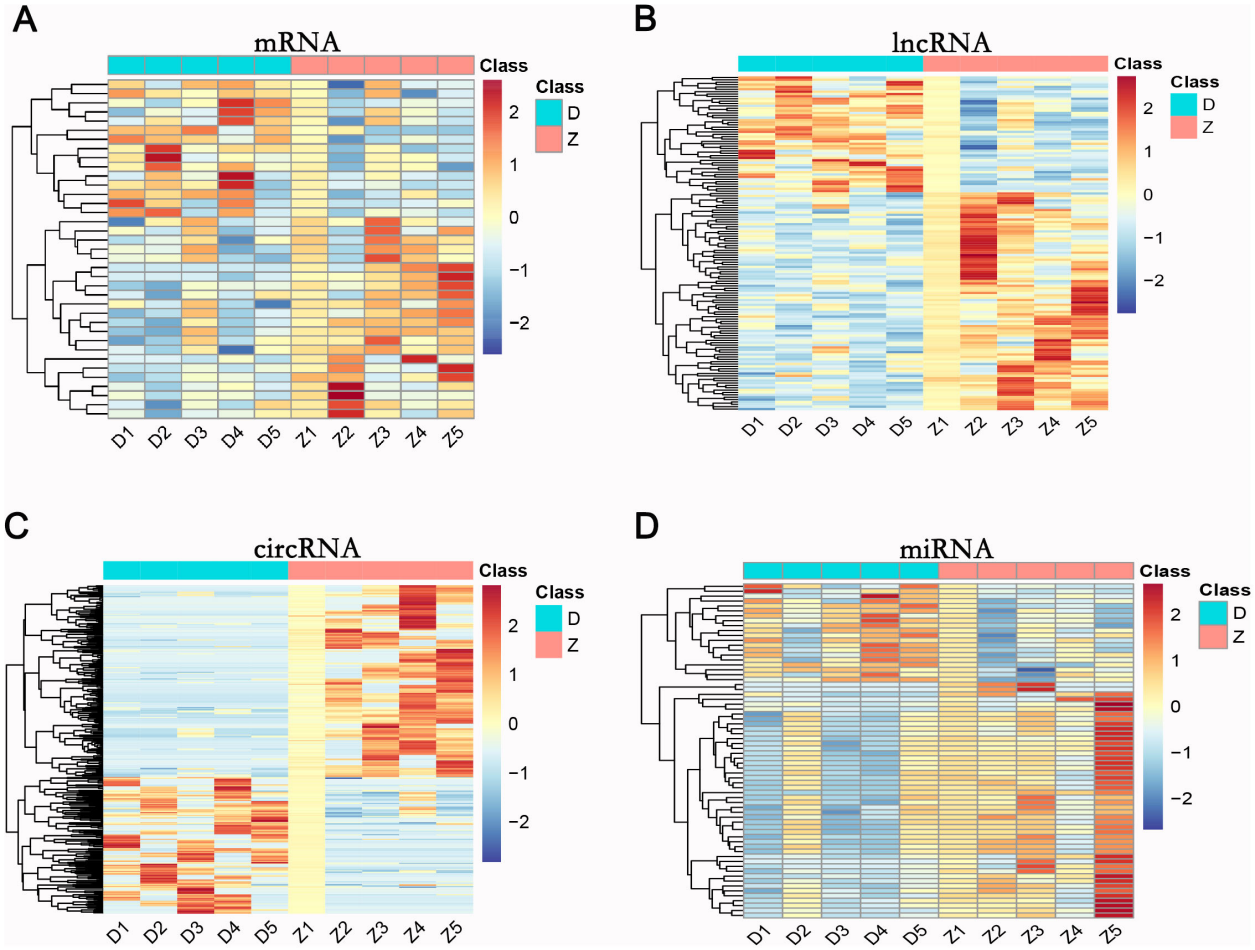
Hierarchical clustering of mRNA **(A)**, lncRNA **(B)**, circRNA **(C)**, and miRNA **(D)** in the T2DM group versus the healthy control group. D1-D5: T2DM group; Z1-Z5: healthy control group. Expression levels range from -2 to 2, indicated by blue (low) to red (high).

Differentially expressed RNAs were obtained by comparing the T2DM group with the control group, using p-value < 0.05 and |log2FC| ≥ 1 as the significant differential expression screening criteria. In T2DM patients, there were 155 upregulated mRNAs and 256 downregulated mRNAs; 280 lncRNAs were upregulated and 220 lncRNAs were downregulated; 148 upregulated circRNAs and 208 downregulated circRNAs; 20 miRNAs were upregulated and 48 miRNAs were downregulated. **Figure 1** is a volcano plot of mRNA (**Figure 1A**), lncRNA (**Figure 1B**), circRNA (**Figure 1C**), and miRNA (**Figure 1D**) showing up- and down-regulation between the T2DM group and the control group. **Figure 2** is a hierarchical clustering heatmap showing differentially expressed mRNA (**Figure 2A**), lncRNA (**Figure 2B**), circRNA (**Figure 2C**), and miRNA (**Figure 2D**) between the T2DM and control groups. Subsequently, we selected the top 10 differentially expressed mRNAs, lncRNAs, circRNAs, and miRNAs, with detailed information in the table below (**Tables 2**–**5**). The complete list of differentially expressed mRNAs, lncRNAs, circRNAs, and miRNAs can be found in **Supplementary Tables 2–5**.

**Table 2:** Top 10 of differently expressed mRNAs in T2DM and healthy control group (sorted by log2FC).

Up-regulated mRNAs			Down-regulated mRNAs		
Gene symbol	log2FC	P-value	Gene symbol	log2FC	P-value
GSTA7P	4.463018544	0.013849931	RP11-497H16.8	-4.818258025	0.000106118
RP11-277J24.1	3.934357007	0.019718059	CH17-224D4.1	-4.540314675	0.00287575
CRIP1P2	3.890676677	0.021346138	RFPL4A	-4.453749798	0.047985258
LINC00854	3.864155714	0.028081441	CEACAM22P	-4.273228073	0.004316207
ANKRD18B	3.863832875	0.019917535	PTGER1	-4.085545256	0.008939968
FBXO40	3.79058236	0.003701683	MYOM2	-3.945077816	1.03E-05
IGKV5-2	3.575937921	0.012685686	RP1-161N10.1	-3.926110925	0.001876678
PLEKHG4B	3.471384094	0.01689961	RP11-1023L17.2	-3.871321983	0.002540518
CHST6	3.389307259	0.011889887	HYDIN	-3.835344575	0.010773094
GALNT9	3.287965906	0.031126279	FGF22	-3.790877813	0.01782695

**Table 3:** Top 10 of differently expressed lncRNAs in T2DM and healthy control group.

Up-regulated lncRNAs			Down-regulated lncRNAs		
lncRNA name	log2FC	P-value	lncRNA name	log2FC	P-value
MSTRG.61365	4.632636116	0.015781113	MSTRG.166130	-5.097280126	0.002557099
MSTRG.233019	4.489204981	0.014310014	MSTRG.303295	-4.616513489	0.003414355
MSTRG.300001	4.342398946	0.005748495	MSTRG.262576	-4.509773286	0.040095649
MSTRG.251833	4.223990129	0.003054521	MSTRG.158980	-4.426551904	0.004695636
MSTRG.182897	4.13841713	0.005048026	MSTRG.201447	-4.416669568	0.021447957
MSTRG.227277	4.135613887	0.012037566	MSTRG.289540	-4.412832532	0.013811347
MSTRG.259007	4.104010207	0.011537004	MSTRG.21063	-4.218333225	0.011639427
MSTRG.232481	4.101708516	0.005182088	MSTRG.17177	-4.123578949	0.015710114
MSTRG.86108	4.084982737	0.00527458	MSTRG.126803	-4.086393135	0.005698791
MSTRG.39484	4.063167617	0.005193353	MSTRG.23713	-4.078047591	0.029197326

**Table 4:** Top 10 of differently expressed circRNAs in T2DM and healthy control group.

Up-regulated circRNAs			Down-regulated circRNAs		
circRNA name	log2FC	P-value	circRNA name	log2FC	P-value
hsa_circ_0019597	4.545299682	0.002360532	hsa_circ_0022608	-4.757520437	0.000953987
hsa_circ_0009210	4.249300453	0.004866427	hsa_circ_0004624	-4.619887339	0.001464771
hsa_circ_0015721	4.214956841	0.005289824	hsa_circ_0022898	-4.590941905	0.001882278
hsa_circ_0015721	4.214956841	0.005289824	hsa_circ_0004023	-4.587461095	0.001610253
hsa_circ_0005063	4.180497971	0.006175107	hsa_circ_0011165	-4.519429408	0.002018218
hsa_circ_0002682	4.166188828	0.006364483	hsa_circ_0007113	-4.474134547	0.002374439
hsa_circ_0028256	4.074806024	0.007944833	hsa_circ_0005941	-4.469147078	0.002316623
hsa_circ_0026738	4.057565289	0.00784581	hsa_circ_0023016	-4.395928332	0.003056259
hsa_circ_0028041	4.054664971	0.007912507	hsa_circ_0006633	-4.365285179	0.003301608
hsa_circ_0008010	4.052534421	0.00835999	hsa_circ_0011196	-4.311748236	0.003581244

**Table 5:** Top 10 of differently expressed miRNAs in T2DM and healthy control group.

Up-regulated miRNAs			Down-regulated miRNAs		
miRNA name	log2FC	P-value	miRNA name	log2FC	P-value
hsa-miR-4449	2.122801	0.000381	hsa-miR-100-5p	-2.36979	7.14E-05
Novel_404	1.96704	0.000702	hsa-miR-410-3p	-1.78487	0.004415
hsa-miR-7113-5p	1.516008	0.007969	hsa-miR-4485-3p	-1.73155	0.003843
hsa-miR-6798-3p	1.340822	0.006259	Novel_452	-1.72737	0.005652
hsa-miR-4525	1.290858	0.041168	Novel_137	-1.66997	0.006452
hsa-miR-1224-3p	1.283284	0.023504	hsa-miR-3613-5p	-1.61149	0.008572
hsa-miR-1914-5p	1.274108	0.034408	hsa-miR-486-3p	-1.60004	0.006626
hsa-miR-6836-5p	1.270451	0.011187	hsa-miR-548ar-3p	-1.50964	0.016516
hsa-miR-6893-3p	1.236879	0.019549	hsa-miR-340-5p	-1.49868	0.001232
hsa-miR-3620-3p	1.223698	0.002084	hsa-miR-27a-5p	-1.47396	0.00212

### GO and KEGG analysis of differentially expressed mRNA, lncRNA, circRNA, and miRNA

**Figure 3:**
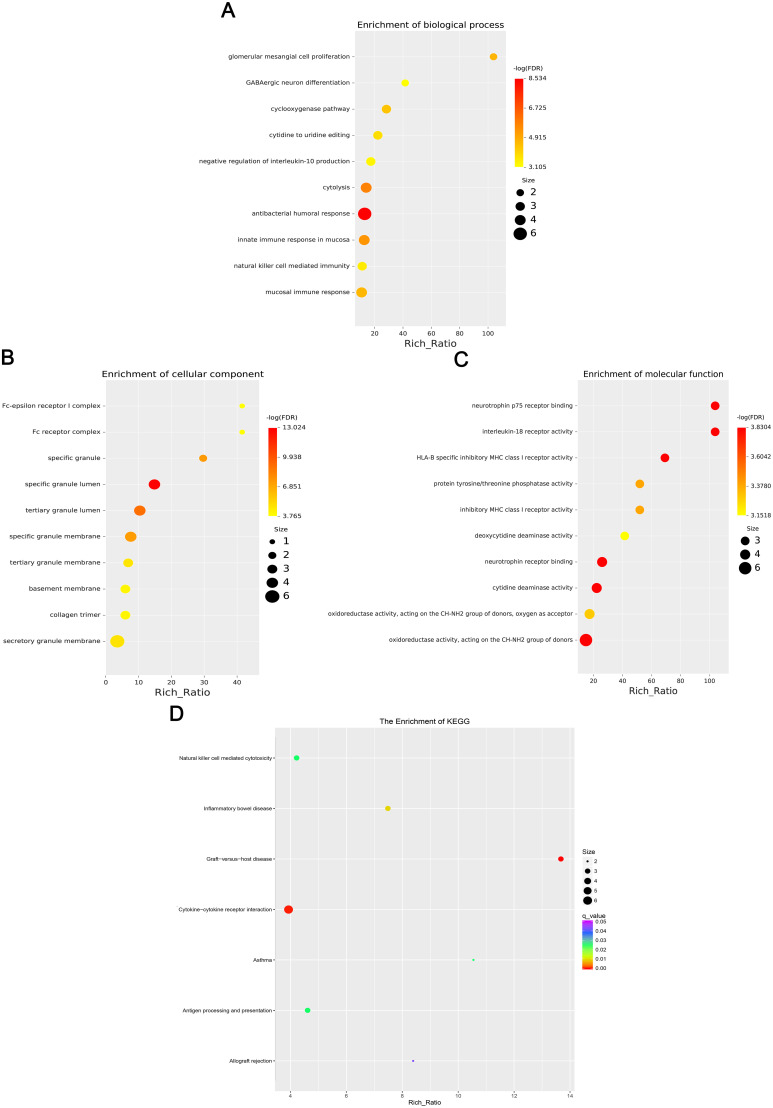
Four scatter plots show the enrichment results of differentially expressed mRNAs. **(A)** Biological processes, **(B)** Cellular components, **(C)** Molecular functions, and **(D)** KEGG enrichment. Dots vary in color and size,indicating different -log(FDR) values and sizes based on a legend. The x-axis shows Rich Ratio for all plots.

**Figure 4:**
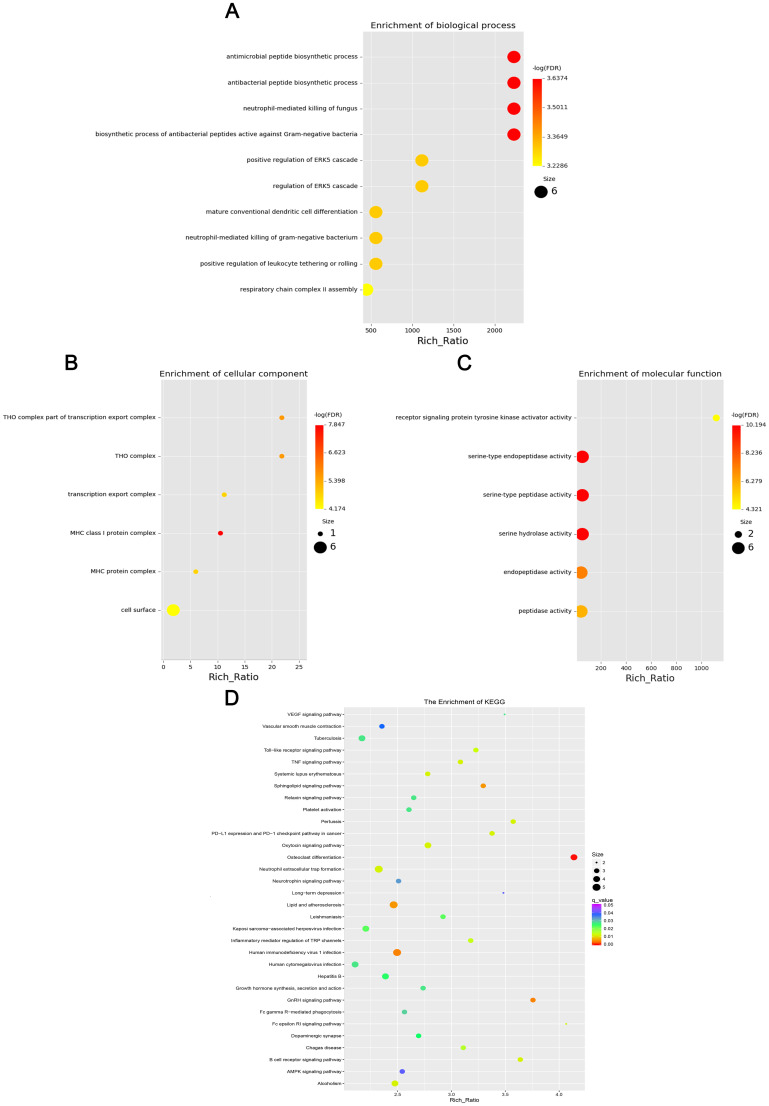
Four scatter plots representing the enrichment of differentially expressed lncRNAs: **(A)** Biological processes with large, red dots for high enrichment, including antimicrobial peptide biosynthetic processes. **(B)** Cellular with smaller, multicolored dots, focusing on THO complex. **(C)** Molecular functions with red and yellow dots, highlighting receptor signaling protein activity. **(D)** KEGG pathways with a color gradient, showing different pathway enrichments like VEGF signaling. Each plot features a Rich Ratio on the x-axis and varying dot sizes indicating significance.

**Figure 5:**
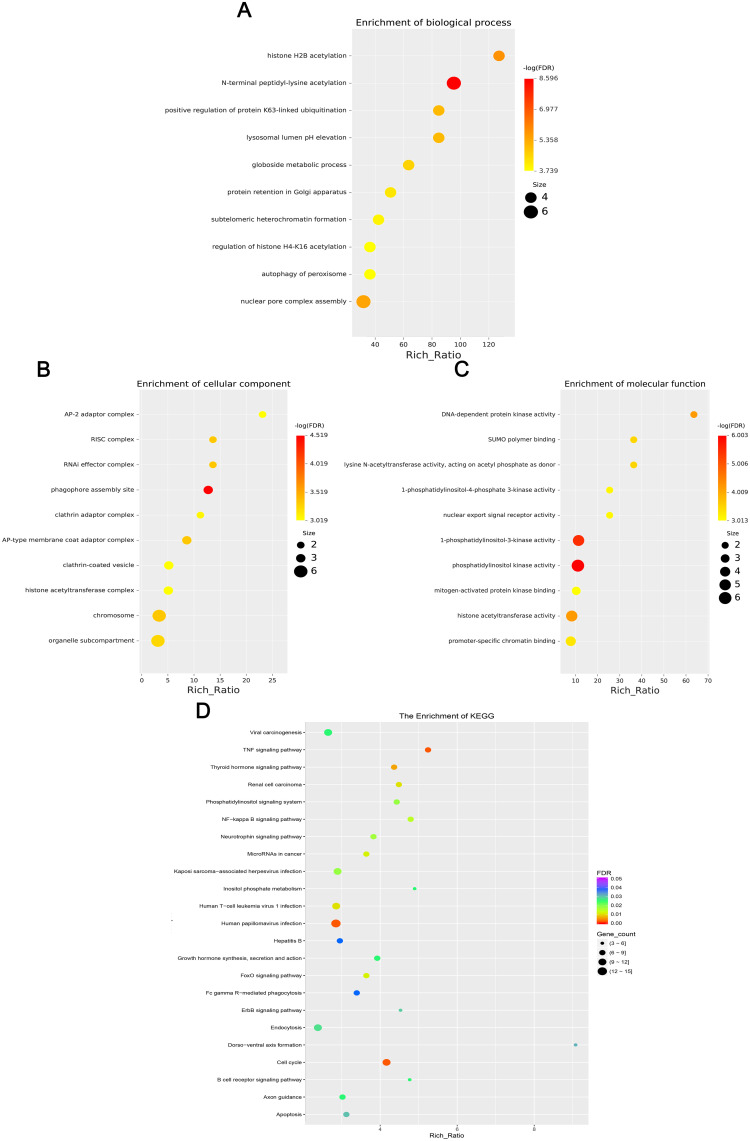
This chart set shows the enrichment analysis of differentially expressed circRNAs. **(A)** Biological processes, **(B)** Cellular components, **(C)** Molecular functions, and **(D)** KEGG enrichment. Dots vary in size and color to indicate rich ratio and significance levels.

**Figure 6:**
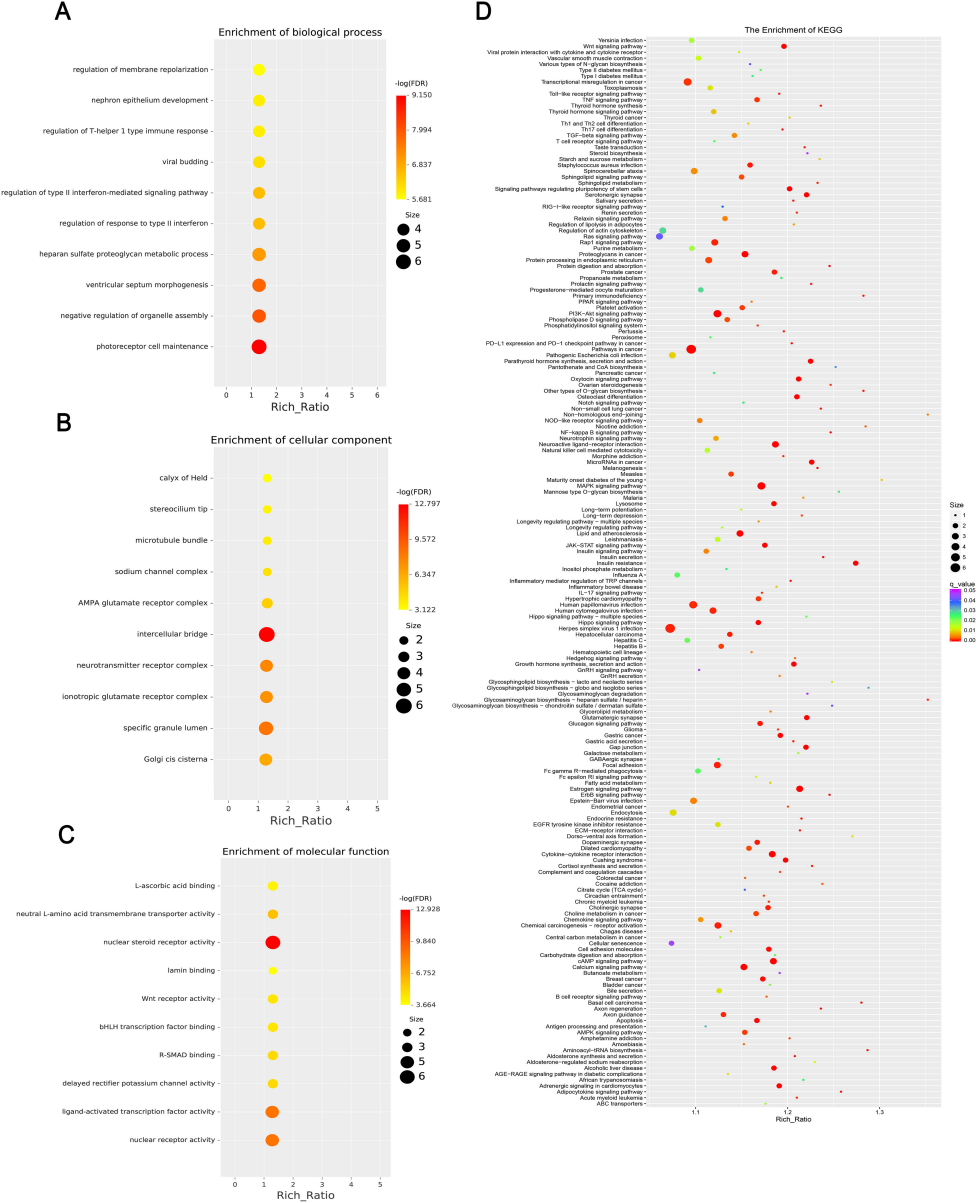
This chart set shows the enrichment analysis of differentially expressed miRNAs. **(A)** Biological processes, highlighting terms such as “regulation of membrane repolarization.” **(B)** Cellular components, enriching terms such as “calyx of Held.” **(C)** Molecular functions, emphasizing terms such as “L-ascorbic acid binding.” and **(D)** KEGG enrichment, including multiple pathways, with significance indicated by color coding and abundance ratio indicated by dot size. Each figure includes a gradient color scale and dots of varying sizes to indicate enrichment significance and abundance.

GO enrichment analysis revealed that the biological processes of the differentially expressed mRNA included mucosal immune response, natural killer cell-mediated immunity, mucosal innate immune response, and antimicrobial humoral response; cellular components involved included secretory granule membranes, specific granule lumen, and third granule lumen; and molecular functions involved included oxidoreductase activity, action on the CH-NH2 donor group, cytidine deaminase activity, neurotrophic factor receptor binding, neurotrophin p75 receptor binding, and interleukin-18 receptor activity (**Figures 3A–C**). In mRNA, pathways highly enriched in the KEGG pathway include cytokine cytokine receptor interactions, graft-versus-host disease, inflammatory bowel disease, etc. (**Figure 3D**). The results of GO functional analysis showed that the main biological processes involved in differentially expressed lncRNA included antimicrobial peptide biosynthesis process, neutrophil-mediated fungal killing process, and regulation of the ERK5 cascade; the cellular components included MHC class I protein complex, THO complex partial transcriptional output complex, and MHC protein complex, etc.; and the molecular functions involved included serine-type endopeptidase activity, serine hydrolase activity, chitin endopeptidase activity, peptidase activity, etc. (**Figures 4A–C**). Among lncRNA, pathways highly enriched in KEGG pathways include osteoclast differentiation, lipids and atherosclerosis, human immunodeficiency virus type 1 infection, GnRH signaling pathway, and sphingolipid signaling pathway (**Figure 4D**). GO functional enrichment analysis of differentially expressed circRNA showed that the major biological processes involved included N-terminal peptidyl lysine acetylation, nuclear pore complex assembly, histone H2B acetylation, etc.; the cellular components involved included phagosome assembly sites, chromosomes, etc.; and for the molecular functions, the main enrichment was in phosphatidylinositol kinase activity, 1-phosphatidylinositol-3-kinase activity, histone acetyltransferase activity, promoter-specific chromatin binding, etc. (**Figures 5A–C**). Pathways in which the KEGG pathway was highly enriched in circRNA included the cell cycle, human papillomavirus infection, TNF signaling pathway, thyroid hormone signaling pathway, and FOXO signaling pathway (**Figure 5D**). Functional enrichment analysis of differentially expressed miRNA showed significant enrichment in biological processes, mainly in secretory granule maturation, dense maturation of core granules, and positive regulation of synaptic vesicle transport. The cellular components involved sodium channel complex, voltage-gated sodium channel complex substance, calyx of Held, semaphorin receptor complex, etc.; and their molecular functions included Brain signaling protein receptor activity, neurotrophin binding, flap endonuclease activity, etc. (**Figures 6A-C**). In miRNA, highly enriched pathways in the KEGG pathway include herpes simplex virus type 1 infection, cancer pathogenesis pathway, actin cytoskeleton regulation, oxytocin signaling pathway, measles, human papillomavirus infection, etc. (**Figure 6D**).

### miRNA-mRNA network analysis

**Figure 7:**
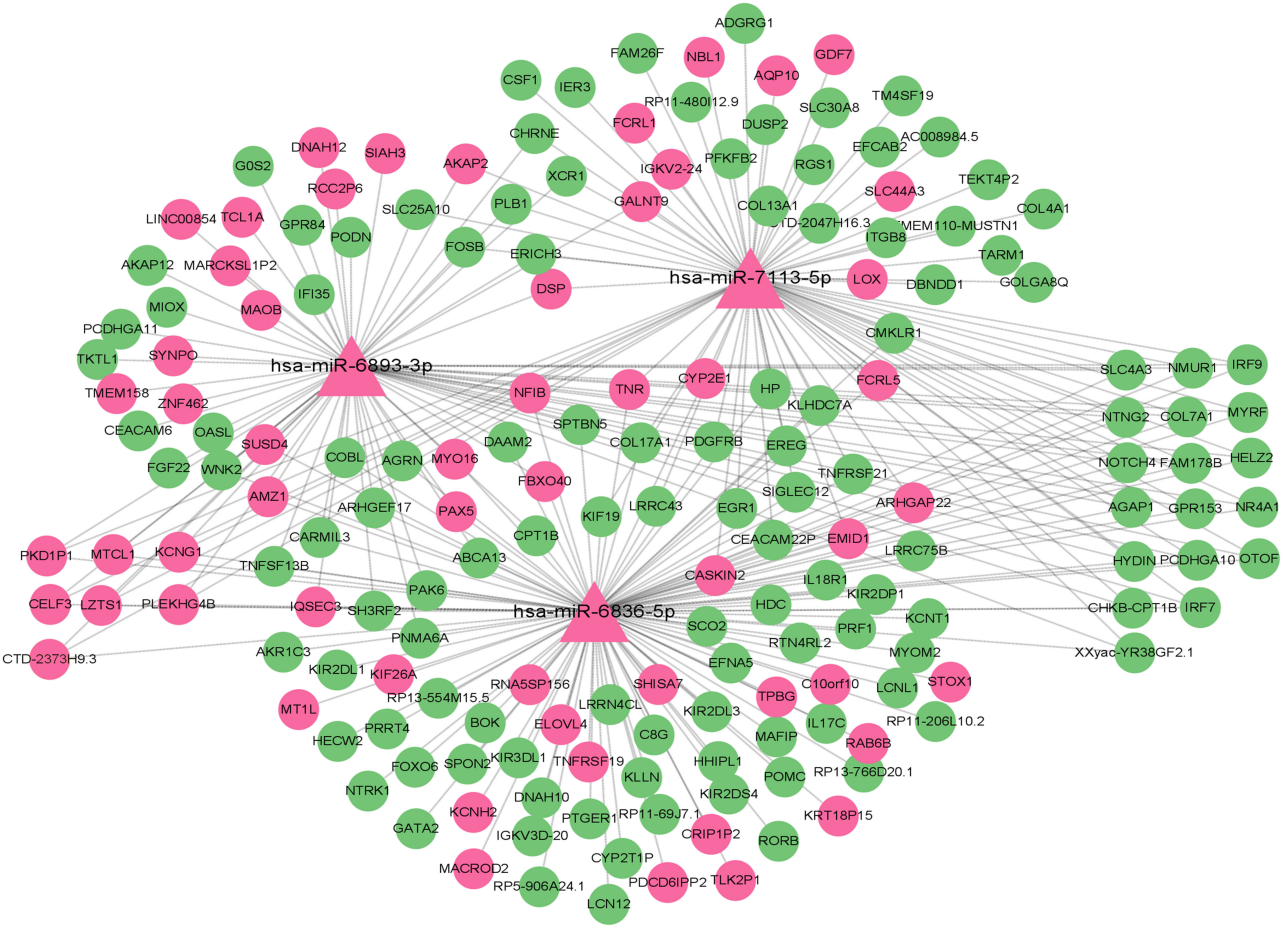
Network diagram showing interactions of three microRNAs, hsa-miR-6893-3p, hsa-miR-7113-5p, and hsa-miR-6836-5p, with various target genes. Nodes are represented by triangles and circles, connected by lines to indicate relationships. Pink indicates up-regulation, and green indicates down-regulation.

To elucidate key regulatory interactions involved in type 2 diabetes, we performed network analysis of differentially expressed miRNAs and their predicted target mRNAs. The miRNA-miRNA interaction network visualized using Cytoscape is shown in **Figure 7**, highlighting important regulatory pairs. We identified the top three miRNAs with the strongest interrelationships. Among the identified miRNAs, miR-6836-5p, miR-7113-5p, and miR-6893-3p emerged as central regulatory factors, jointly targeting 25 key genes, including NR4A1, IRF7, and SLC4A3. These genes are associated with insulin secretion, fat metabolism, and inflammatory responses.

### Construction of ceRNA network mediated by differentially expressed circRNA

**Figure 8:**
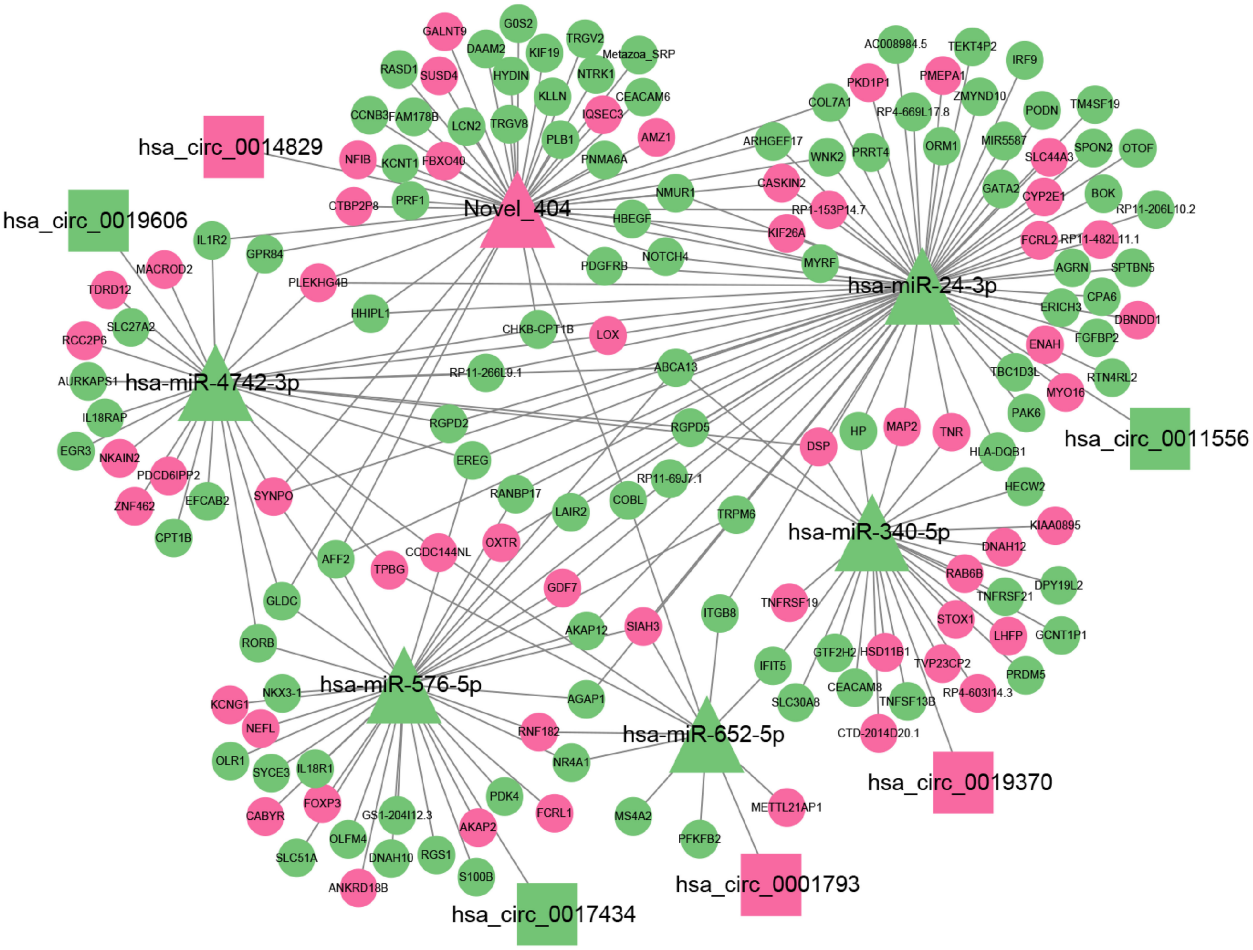
Network diagram showing relationships between circRNA, miRNA, and mRNA. Triangular nodes represent miRNA; rectangular nodes represent circRNA; circular nodes represent mRNA; pink represents up-regulation; green nodes represent down-regulation. Lines connecting nodes indicate interactions. Key tags include hsa_circ_0014829, hsa-miR-4742-3p, Novel 404, etc.

ceRNA has been a hot topic of research in recent years. To study the ceRNA regulation of differentially expressed circRNAs in 2TDM, we constructed a ceRNA network mediated by differentially expressed circRNAs. Ultimately, a ceRNA network was constructed based on 6 circRNAs (3 upregulated and 3 downregulated), 6 miRNAs, and 156 mRNAs, resulting in 6 pairs of circRNA-miRNA and 216 pairs of miRNA-mRNA relationships (**Figure 8**). Among these, hsa_circ_0014829 and Novel_404, hsa_circ_0019606 and hsa-miR-4742-3p, hsa_circ_0017434 and hsa-miR-576-5p, hsa_circ_0001793 and hsa-miR-652-5p, hsa_circ_0019370 and hsa-miR-340-5p, and hsa_circ_0011556 and hsa-miR-24-3p. Among these, Novel_404 was significantly upregulated in 2TDM, while hsa-miR-4742-3p, hsa-miR-652-5p, hsa-miR-24-3p, hsa-miR-576-5p, and hsa-miR-340-5p were significantly downregulated in 2TDM. The sequence information of the key circRNAs is shown in **Table 6**.

**Table 6:** Sequence information of key lncRNAs.

circRNA name	log2FC	P-value	Up/down	Interaction position	Combined miRNA
hsa_circ_0011556	-4.14512	0.00588	down	chr19:54633016-54697945: +	hsa-miR-24-3p
hsa_circ_0019606	-2.64273	0.03324	down	chr3:18463480-18527326: +	hsa-miR-4742-3p
hsa_circ_0014829	3.18827	0.04759	up	chr20:34732559-34743341: -	Novel_404
hsa_circ_0017434	-4.20563	0.00533	down	chr2:230436381-230450255: +	hsa-miR-576-5p
hsa_circ_0001793	3.56464	0.02446	up	chr11:16014942-16111926: -	hsa-miR-652-5p
hsa_circ_0019370	1.39288	0.03862	up	chr3:17009674-17014911: +	hsa-miR-340-5p

### Construction of ceRNA network mediated by differentially expressed lncRNA

**Figure 9:**
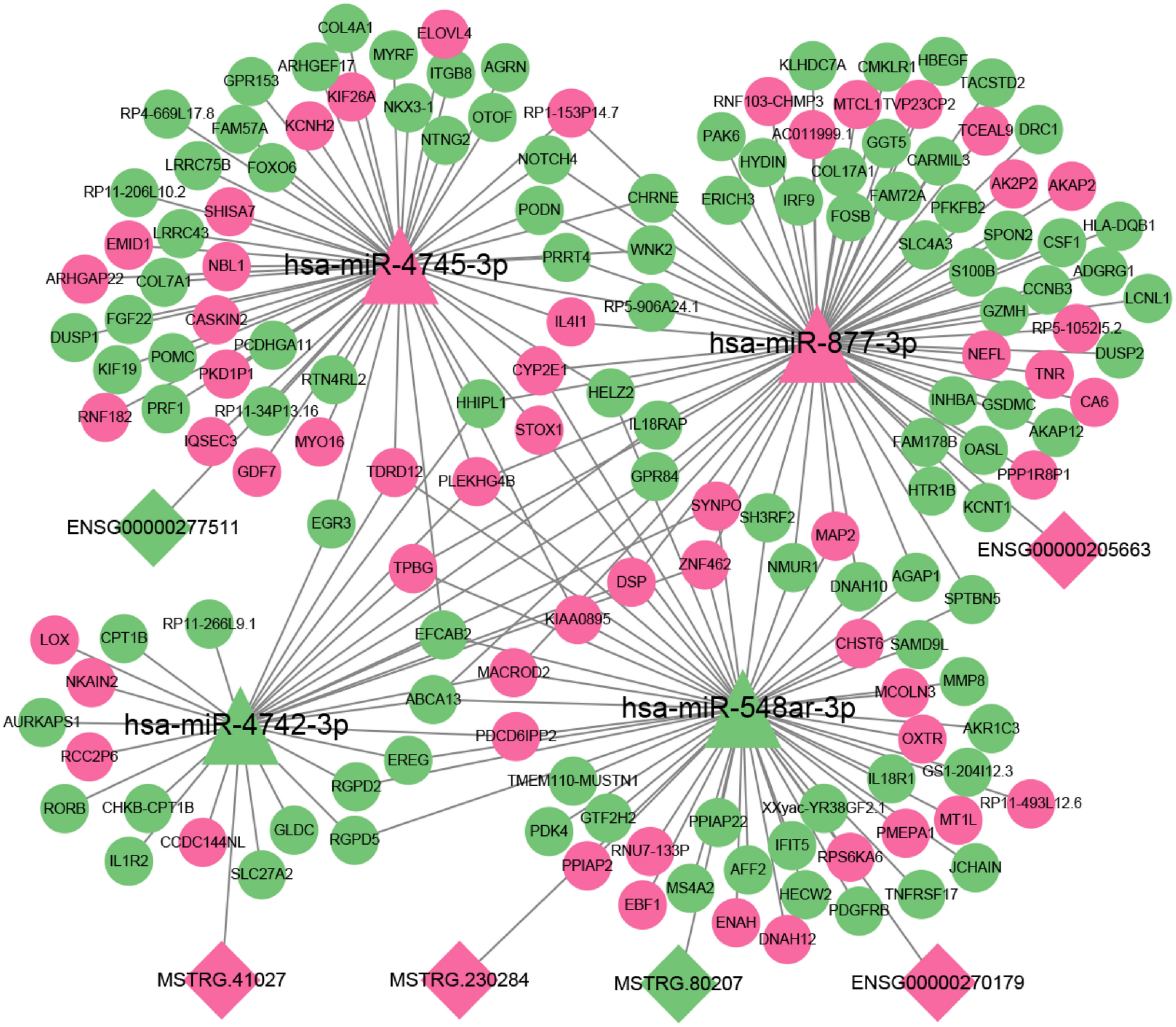
A complex network diagram illustrating the interactions between miRNAs (hsa-miR-4745-3p, hsa-miR-877-3p, hsa-miR-4742-3p, hsa-miR-548ar-3p) interact with lncRNAs and mRNAs. Triangular nodes represent miRNA; diamond nodes represent lncRNA; and circular nodes represent mRNA. Pink indicates up-regulation, and green indicates down-regulation. Lines connecting nodes illustrate potential regulatory relationships.

To further explore the role of lncRNAs in the pathogenesis of 2TDM, we constructed a lncRNA-mediated ceRNA network using differentially expressed lncRNAs in 2TDM and predicted interactions between lncRNAs, mRNAs, and miRNAs using TargetScan and miRanda, generating 6 pairs of lncRNA-miRNA relationships and 205 pairs of miRNA-mRNA relationships (**Figure 9**). Among these, three lncRNAs can establish relationships with hsa-miR-548ar-3p. In addition, ENSG00000277511 and hsa-miR-4745-3p, ENSG00000205663 and hsa-miR-877-3p, and MSTRG.41027 and hsa-miR-4742-3p only establish mutual relationships. Three lncRNAs (MSTRG.80207, MSTRG.230284, and ENSG00000270179) can act as ceRNAs to competitively bind with hsa-miR-548ar-3p and regulate the function of 55 mRNAs. In this network, hsa-miR-877-3p and hsa-miR-4745-3p were significantly upregulated, while hsa-miR-548ar-3p and hsa-miR-4742-3p were significantly downregulated. Additionally, circRNA hsa_circ_0019606 was found to be positively correlated with hsa-miR-4742-3p, while lncRNA MSTRG.41027 was negatively correlated with hsa-miR-4742-3p. The sequence information of the key lncRNAs is shown in **Table 7**.

**Table 7:** Sequence information of key circRNAs.

lncRNA name	log2FC	P-value	Up/down	Interaction position	Combined miRNA
MSTRG.80207	-1.06442	0.01164	down	chr13:114241049-114268489: -	hsa-miR-548ar-3p
MSTRG.41027	1.17097	0.02024	up	chr10:124574512-124576602: +	hsa-miR-4742-3p
MSTRG.230284	1.42112	0.00193	up	chr5:177904944-177908700: +	hsa-miR-548ar-3p
ENSG00000277511	-1.30361	0.00916	down	chr17:32127595-32128454: +	hsa-miR-4745-3p
ENSG00000270179	1.25808	0.00867	up	chr11:113368478-113369117: +	hsa-miR-548ar-3p
ENSG00000205663	1.89203	0.03396	up	chrX:3891438-3920746: -	hsa-miR-877-3p

## Discussion

With the development of molecular biotechnology, ncRNA has been found to play important roles in various biological processes (22). Studies have found that the abnormal expression of lncRNA or circRNA is related to the occurrence and development of diabetes, such as XLOC-013310 (23), cirS-7, and circHIPK3 (13). However, the underlying mechanisms and biomarkers of T2DM still deserve further exploration. Therefore, we sought to characterize the molecular features of the disease by analyzing the differential expression of mRNA, lncRNA, circRNA, and miRNA in the T2DM group versus the healthy control group and attempted to further our understanding of the mechanisms.

This study is an exploratory phase study aimed at screening potential key molecular targets for type 2 diabetes mellitus (T2DM) through high-throughput sequencing. Strict screening criteria can reduce individual heterogeneity and improve the detection efficiency of small samples. Similar strategies have been used in various biomarker screening studies. For example, Yang F et al. identified differentially expressed RNAs in RNA-seq of three pairs of T2DM samples and explored the molecular mechanisms underlying T2DM using a ceRNA network established by circRNA and lncRNA (24). R et al. identified candidate molecules for subsequent validation in RNA-seq of three pairs of ischemic strokes, screening five circRNAs and 13 lncRNAs that can act as ceRNAs to compete with miRNAs in co-expression networks and may play an important role in hemorrhagic transformation (8). This article focuses on studying changes in the peripheral blood transcriptome in T2DM. Previous studies have shown that peripheral blood (white blood cells/PBMCs) can effectively reflect changes in inflammation, metabolism, and immune pathways associated with T2DM. Furthermore, multiple studies have confirmed that the leukocyte transcriptome can identify T2DM-specific gene markers and is significantly correlated with clinical parameters (such as HbA1c) (25). In addition, changes in the leukocyte transcriptome are highly correlated with insulin resistance and inflammatory pathways (26). Blood RNA-seq technology can identify genes associated with insulin resistance, and the study also highlights the potential value of miRNAs as pathological physiological markers for distinguishing T2DM subtypes (27). Therefore, this study utilized high-throughput sequencing technology to perform whole-transcriptome RNA sequencing on peripheral blood samples from five pairs of T2DM patients and healthy controls, and analyzed the differential expression profiles of mRNA, lncRNA, circRNA, and miRNA between the T2DM and healthy control groups. Compared with the control group, the T2DM group had 411 differentially expressed mRNAs (155 up-regulated and 256 down-regulated), 500 differentially expressed lncRNAs (280 up-regulated and 220 down-regulated), 356 differentially expressed circRNAs (148 up-regulated and 208 down-regulated), and 68 differentially expressed miRNAs (20 up-regulated and 48 down-regulated). However, the expression levels of the key differentially expressed RNAs require further validation through qRT-PCR experiments with a larger sample size. Utilizing these differentially expressed genes and ribonucleic acids provides possible opportunities for early diagnosis and intervention in T2DM.

Previous studies have found that the mTOR signaling pathway and the lysosomal pathway are closely related to type 2 diabetes. Insulin activates mTORC1 by inducing the dissociation of the TSC complex and lysosomes (28). However, excessive activation of mTORC1 leads to insulin resistance (29). Therefore, we analyzed by GO and KEGG enrichment to obtain the biological functions and potential pathways of these differentially expressed mRNA, lncRNA, circRNA and miRNA. It was found that cytokine-cytokine receptor interaction, graft-versus-host disease, inflammatory bowel disease, lipid and atherosclerosis, human immunodeficiency virus type 1 infection, GnRH signaling pathway, sphingolipid signaling pathway, cell cycle, TNF signaling pathway, FOXO signaling pathway, etc. played an important role in T2DM. The terms immune response, 1-phosphatidylinositol-3-kinase activity, oxidoreductase activity, interleukin-18 receptor activity, antimicrobial peptide biosynthesis process, neutrophil-mediated fungal killing process, and regulation of ERK5 cascade were enriched in the gene list. T2DM is a generalized metabolic disorder characterized not only by hyperglycemia but also by dyslipidemia, and these lead to elevated cardiovascular risk (30). Their dyslipidemia is usually characterized by reduced levels of HDL cholesterol and elevated levels of atherosclerosis-induced lipids or lipoproteins (31). Sphingolipids maintain the structural integrity of cell membranes and regulate many key cellular processes through signaling and gene regulation, which have been implicated in the onset and progression of a variety of diseases, including diabetes, inflammatory bowel disease, and asthma (32). TNF is a multifunctional pro-inflammatory cytokine that triggers a signaling process upon binding to its receptor, which activates the NF-κB signaling pathway to promote an inflammatory response (33). TNF also activates the mitogen-activated protein kinase (MAPK) pathway to enhance inflammatory responses (34). TNF-α affects glucose metabolism by inhibiting insulin action through serine phosphorylation of IRS proteins (35). 1-phosphatidylinositol-3-kinase (PI3K) activity was found to be significantly enriched in T2DM, and reduced activity of PI3K, a key enzyme in insulin signaling, may lead to insulin resistance and elevated blood glucose. It is well known that the PI3K/Akt signaling pathway is involved in the regulation of glucose metabolism, cell proliferation, apoptosis, and other processes (36). FOXO protein is a transcription factor that plays an important role in cell proliferation and apoptosis, metabolism, and oxidative stress (37). Many studies have shown that in type 2 diabetes, FOXO1 is involved in glucose and lipid metabolism, insulin resistance, and β-cell proliferation, differentiation, and apoptosis, making it an important target for potential therapeutic intervention (38). FOXO1 normally remains inactive in pancreatic β-cells under normal conditions but is activated in response to hyperglycemia, and loss of FOXO1 function in β-cells has been found to correlate with reduced insulin secretion (39). Cai Z et al. found that FoxO1 protects pancreatic β cells from damage induced by low levels of reactive oxygen species (ROS) by upregulating superoxide dismutase (SOD) (40). FOXO1 plays a dual role in pancreatic β-cell proliferation and apoptosis. It can inhibit cell apoptosis by activating antioxidant genes, and it can also induce pro-apoptotic genes to promote cell apoptosis (41). FOXO1 also participates in regulating cellular stress responses and proliferation, thereby affecting β-cell differentiation and survival. Decreased phosphorylation of FOXO1 affects its protein levels and transcriptional activity, activating genes related to gluconeogenesis, thereby increasing glucose production and exacerbating hyperglycemia in insulin-resistant cells (42). Kamal MM et al. found that silencing FOXO-1 enhanced the insulin-producing cell generation of adipose-derived mesenchymal stem cells and could be used for the treatment of diabetes (43). FOXO1 promotes hepatic glucose production and regulation of lipid metabolism, which, in the presence of insulin resistance, leads to hyperglycemia and dyslipidemia (44). It is known that the FOXO signaling pathway mediated by FOXO factor plays an important role in the pathogenesis of T2DM. Hyperglycemia drives neutrophil production and mobilization, which promotes the development and progression of diabetic complications (45). In our study, genes and ribonucleic acids from T2DM patients were significantly enriched in immune response, TNF signaling pathway, FOXO pathway, and sphingolipid signaling pathway. Therefore, we speculate that differentially expressed genes and ribonucleic acids in T2DM may affect the development of T2DM through pathways related to glucose metabolism, lipid metabolism, and immune response.

In this study, we constructed a miRNA-mRNA regulatory network and identified several key target genes, including NR4A1 and IRF7. NR4A1 is a member of the Nr4a nuclear receptor superfamily that regulates inflammatory responses (46). Research has found that NR4A1 is an attractive target for improving insulin resistance and preventing and treating T2DM and metabolic diseases (47). IRF7, interferon regulatory factor 7, belongs to the interferon regulatory factor family and is one of the main regulatory factors for type I interferon production (48). IRF7 deficiency can prevent diet-induced obesity and insulin resistance (49). ceRNA is a class of RNA molecules (including lncRNA, circRNA, pseudogenes, mRNA, etc.) that can competitively bind to miRNAs through shared miRNA response elements (MREs), thereby regulating the inhibitory effect of miRNAs on their target genes and forming a complex post-transcriptional regulatory network. Therefore, we constructed circRNA-miRNA-mRNA and lncRNA-miRNA-mRNA ceRNA regulatory network diagrams based on transcriptomic data. Six circRNAs and six lncRNAs were selected as ceRNA to compete with miRNA in the co-expression network, which are related to the pathogenesis of T2DM by regulating the function of mRNA in the network. We screened three significantly up-regulated miRNAs (Novel_404, hsa-miR-877-3p,and hsa-miR-4745-3p) and six significantly down-regulated miRNAs (hsa-miR-4742-3p, hsa-miR-652-5p, hsa-miR-24-3p, hsa-miR-340-5p, hsa-miR-576-5p, hsa-miR-548ar-3p). And we found that the relationship between Novel_404, hsa-miR-4745-3p, hsa-miR-4742-3p, and hsa-miR-548ar-3p and the pathogenesis of the disease had not been reported before. Previous studies have found that the expression of miR-877-3p in UExos is significantly upregulated in patients with diabetic nephropathy (50). Downregulation of hsa-miR-877 in osteosarcoma and ovarian cancer tissues (51, 52); similarly, hsa-miR-877-3p is down-regulated in esophageal squamous cell carcinoma expression as a tumor suppressor gene, and its down-regulation is associated with poor prognosis in esophageal squamous cell carcinoma (53). Liu S et al. found that the impaired miR-652-5p/Tigar axis inhibits glycolysis, thereby slowing the growth of acute T-lymphoblastic leukemia (T-ALL) cells, and suggests that miR-652-5p may serve as a novel potential drug target for the treatment of T-ALL (54). MiR-24-3p is elevated in the serum of children with T1DM (55), and its overexpression inhibits β-cell proliferation and insulin secretion (56). Compared with the control group, the level of hsa-miR-24-3p expressed in T2DM patients was reduced (57); this is consistent with our findings. Research has found that the improvement of blood glucose status in T2DM patients treated with metformin is directly related to the decrease in hsa-miR-24-3p levels (58). Zhu Y et al. found that miR-340-5p is up-regulated in diabetes cardiomyopathy, which can target therapeutic intervention (59). Expression levels of miR-576-5p were significantly elevated in thyroid cancer tissues. miR-576-5p promotes the proliferation of thyroid-like cancers through the MAPK4-AKT pathway (60). Through these network diagrams, we can more clearly identify key regulatory nodes and further verify the important role of these non-coding RNAs in the onset and development of diseases. At the same time, these analysis results provide strong evidence for subsequent functional experiments.

This study still has some limitations. First, the sample size included in this study is limited, which may affect the accuracy of the extrapolation of the research results. Furthermore, this study is only a preliminary exploration and requires further validation of transcriptomic sequencing results through *in vivo* or *in vitro* experiments. Although our RNA sequencing analysis identified a significant differentially expressed gene profile, independent technical validation (such as qRT-PCR or Nanostring) remains critical for translational applications. qRT-PCR can validate the reliability of differentially expressed genes through highly specific primers and dynamic detection. Additionally, for low-abundance transcripts, the sensitivity of qRT-PCR can compensate for insufficient sequencing depth, providing precise molecular targets for subsequent mechanistic studies. Future studies will expand qRT-PCR validation to more cohorts and utilize β-cell models to perform functional characterization of priority targets (e.g., Novel_404, hsa-miR-877-3p, hsa-miR-4745-3p, etc.).

## Conclusions

The expression levels of circRNA, miRNA, lncRNA, and mRNA differ in patients with T2DM. Enrichment analysis revealed that the tumor necrosis factor (TNF) signaling pathway, FOXO pathway, and phosphoinositide-3 kinase (PI-3K)-mediated signaling pathway are closely associated with glucose metabolism. Additionally, through the constructed ceRNA network, it is evident that the mutual regulation among mRNA, lncRNA, circRNA, and miRNA may play a role in the development of T2DM.

## Data Availability

The data presented in this study are deposited in the NCBI BioProject repository, accession number PRJNA1307011, https://www.ncbi.nlm.nih.gov/bioproject/PRJNA1307011.
