# Supplementary material for: Differential expression and correlation analysis of whole transcriptome for type 2 diabetes mellitus
Source: Front Endocrinol (Lausanne). 2025 Aug 29;16:1541261. doi: 10.3389/fendo.2025.1541261 (PMC12425763; doi:10.3389/fendo.2025.1541261)
Supplement: Supplementary file 1 [file Table1.docx]

supplementary material：

| **Table S1：Data filtering of circRNA, lncRNA and mRNA** | | | | | |
| --- | --- | --- | --- | --- | --- |
| CircRNA | | | | | |
| sample | raw_reads | clean_reads | clean_rate(%) | Clean Q30(%) | Clean GC (%) |
| Z1 | 104580024 | 97370628 | 93.11 | 92.00 | 49.12 |
| Z2 | 100953036 | 95551370 | 94.65 | 92.22 | 47.48 |
| Z3 | 88201834 | 83143362 | 94.26 | 93.18 | 50.01 |
| Z4 | 106168838 | 95994314 | 90.42 | 91.62 | 50.63 |
| Z5 | 101218220 | 94235596 | 93.10 | 91.72 | 49.08 |
| D1 | 79376472 | 75818172 | 95.52 | 92.71 | 48.21 |
| D2 | 80422494 | 75889210 | 94.36 | 91.78 | 48.77 |
| D3 | 96738868 | 92141250 | 95.25 | 92.99 | 47.47 |
| D4 | 88239320 | 82224200 | 93.18 | 93.05 | 50.11 |
| D5 | 100978016 | 93285246 | 92.38 | 91.32 | 49.31 |
| LncRNA | | | | | |
| Z1 | 104580024 | 97370628 | 93.11 | 92.00 | 49.12 |
| Z2 | 100953036 | 95551370 | 94.65 | 92.22 | 47.48 |
| Z3 | 88201834 | 83143362 | 94.26 | 93.18 | 50.01 |
| Z4 | 106168838 | 95994314 | 90.42 | 91.62 | 50.63 |
| Z5 | 101218220 | 94235596 | 93.10 | 91.72 | 49.08 |
| D1 | 79376472 | 75818172 | 95.52 | 92.71 | 48.21 |
| D2 | 80422494 | 75889210 | 94.36 | 91.78 | 48.77 |
| D3 | 96738868 | 92141250 | 95.25 | 92.99 | 47.47 |
| D4 | 88239320 | 82224200 | 93.18 | 93.05 | 50.11 |
| D5 | 100978016 | 93285246 | 92.38 | 91.32 | 49.31 |
| miRNA | | | | | |
| Z1 | 104580024 | 97370628 | 93.11 | 92.00 | 49.12 |
| Z2 | 100953036 | 95551370 | 94.65 | 92.22 | 47.48 |
| Z3 | 88201834 | 83143362 | 94.26 | 93.18 | 50.01 |
| Z4 | 106168838 | 95994314 | 90.42 | 91.62 | 50.63 |
| Z5 | 101218220 | 94235596 | 93.10 | 91.72 | 49.08 |
| D1 | 79376472 | 75818172 | 95.52 | 92.71 | 48.21 |
| D2 | 80422494 | 75889210 | 94.36 | 91.78 | 48.77 |
| D3 | 96738868 | 92141250 | 95.25 | 92.99 | 47.47 |
| D4 | 88239320 | 82224200 | 93.18 | 93.05 | 50.11 |
| D5 | 100978016 | 93285246 | 92.38 | 91.32 | 49.31 |
